# Medical Schools

**Published:** 1865-10

**Authors:** 


The following from an editorial in a recent number of the "Medical and Surgical Reporter" is applicable as well to the dental as the medical student. The courses in our dental schools *are* too short:

This is what we should advise every student to do; if at all possible, attend three courses of lectures. During the first course pay particular attention to anatomy, chemistry, materia medica and physiology. Master these branches perfectly, they form the corner-stones of medical science. The second year throw yourself with all your energy upon general and special pathology, surgery and midwifery, paying attention to the other branches only sufficient to retain what has already been learned. The third course you can hear every branch with both pleasure and profit; take no notes, however, of didactic lectures, but let your main business be to study and write up clinical and hospital cases, and be a *fixture* at all college and hospital clinics.

A plan more or less similar to the above, at any rate distributing the study of medicine over a larger space of time, has for years been and is yet followed by many students, and the necessity of some general action among the faculties of our medical schools, tending to the same end, will, we have no doubt, make itself felt more strongly every year. There can certainly be no impropriety in our calling the attention of the profession to the fact that an increase in the number of teachers, and addition to the branches to be taught demand an extension of the period of instruction. It has long been admitted, generally, that it is, under any circumstances, too short.

